# Late entry to antenatal care in New South Wales, Australia

**DOI:** 10.1186/1742-4755-3-8

**Published:** 2006-08-18

**Authors:** Lieu Thuy Thi Trinh, George Rubin

**Affiliations:** 1 Centre for Health Services Research, University of Sydney and Western Sydney Area Health Services, Level 3, Administration Block, Westmead Hospital, Westmead, NSW 2145, Australia

## Abstract

**Aims:**

This study aimed to assess the prevalence of women who entered antenatal care (ANC) late and to identify factors related to the late entry to ANC in New South Wales (NSW) in 2004.

**Methods:**

The NSW Midwives Data Collection contained data of 85,034 women who gave birth in 2004. Data were downloaded using SAS and transferred to STATA 8.0. Entering ANC after 12 weeks of gestation was classified as late. The Andersen Health Seeking Behaviour Model was used for selection and analyses of related factors. Regression and hierarchical analyses were used to identify significant factors and their relative contributions to the variation of pregnancy duration at entry to ANC.

**Results:**

41% of women commenced ANC after 12 weeks of gestation. Inequality existed between groups of women with predisposing characteristics and enabling resources contributed more to the variation in pregnancy duration at entry to ANC than needs. The groups of women with highest risk were teenagers, migrants from developing countries, women living in Western Sydney, Aboriginal and Torres Strait Islanders, women with three or more previous pregnancies and heavy smokers. The high risk groups with largest number of women were migrants from developing countries and women living in Western Sydney.

**Conclusion:**

A large number of women in NSW entered ANC late in their pregnancies. Efforts to increase early entry to ANC should be targeted on identified high risk groups of women.

## Background

### Pregnancy duration at entry to antenatal care in Australia

Early entry to antenatal care (ANC) is important for early detection and treatment of adverse pregnancy related outcomes. The World Health Organization (WHO) recommends that pregnant women in developing countries should seek ANC within the first 4 months of pregnancy [1]. In developed countries such as the United Kingdom and the United States, ANC is recommended within the first 12 weeks of pregnancy [2,3].

The WHO recently recommended a reduction in the number of ANC visits in developed countries because of evidence suggesting that having fewer ANC visits does not affect the outcomes of care, other than women's satisfaction levels [4,5]. However, women are still advised to attend ANC early and even earlier than previously recommended. For example, the United State Public Health Service Expert Panel on Prenatal Care recommends a first visit in the first eight weeks of pregnancy and, ideally, before conception in order to identify and treat health conditions that could affect the foetus [6].

Australia does not have a national guideline on ANC, however a review of 80 local guidelines showed that 79% of these guidelines are similar to the one from the United Kingdom [7] which recommends first visit within 12 weeks. The New South Wales (NSW) Health Department Midwives Data Collection (MDC) uses the cut off point of 20 weeks for classifying late ANC. This cut off point is later than all recommendations for developing countries [1], developed countries [2,3] and the majority of other states in Australia [7,8].

A majority of pregnant women in Australia enter ANC early but a significant number of women do not do so. Twenty percent of pregnant women in Victoria in 1997–1998 had first visit after three months [8] and 13% of women in NSW in 1998 attended ANC after 20 weeks of gestation [9].

There have been many studies on factors relating to late entry to ANC in the world. The related factors include place of residence [10-12], ethnicity [13], age [11], education [12,14,15], employment status[13,16], parity [11-13,15], intention to get pregnant [10,13], use of contraceptive method [13], economic status [10,13,14], health insurance [10-12,14,17] and travel time [10].

On the other hand, there is limited information on the factors related to late entry to ANC in Australia. One study in NSW reported that women from non English speaking background, Aboriginal and Torres Strait origin were more likely to enter ANC after 20 weeks than others [18]. However, there have been no studies that used the cut off point of 12 weeks and a theoretical health seeking behaviour model as a framework for selection and analysis of related factors.

### The Andersen Health Seeking Behaviour Model

The Andersen Health Seeking Behaviour Model [19,20] assumes that health seeking behaviour is the result of interaction between characteristics of individuals, population and the surrounding environment. The model consists of several main components: predisposing characteristics (eg. age, race), enabling resources (eg. health insurance), needs (eg. being sick or having further complications), personal health care behaviour (eg. exercise), outcomes (eg. satisfaction with the health services) and environment (eg. health care policies). Inequality exists if factors other than needs, such as enabling resources, are the dominants of health care utilisation. That means people utilise health services mainly because their resources allow them to, but not because of the severity of their sickness.

The model has been widely used in studying factors related to utilisation of different health care services, which includes 139 studies reviewed by Phillips el al [21]. The model has also been used in several studies on ANC in other countries [11,12,14,16,22].

### Aims

The aims of this study were to assess the proportion of pregnant women who entered ANC late in NSW during calendar year 2004 and to identify factors related to late entry.

### Significance

The results enable the targeting of ANC resources to improve maternal and infant health in NSW.

## Methods

### Data

The NSW Health Department has established a data base library called the Health Outcomes Information and Statistical Toolkit (HOIST) in 1988. Reporting to HOIST is compulsory. One of the databases in HOIST is the NSW MDC [23] which contains information on mothers and babies. The MDC 2004 recorded pregnancy duration at entry to ANC in weeks and a number of potential related factors.

### Outcome variables

Weeks of pregnancy duration at first ANC visit was treated as a continuous variable and was also classified into late and early entry to ANC. Late ANC was defined as entering ANC after 12 weeks and early as 12 weeks or earlier. This cut off point was used because it is the common recommendation for developed countries [2,3] and the majority of Australia [7]. The proportions of women who failed to meet the recommendation of the WHO for developing countries (4 months or 17 weeks) [1] and the recommendation used for the NSW Midwife Data Collection (20 weeks) were also reported for comparison.

### Explanatory variables

The first column of Table 1 presents nine potential explanatory variables and their detailed classifications. These variables reflect four main components of the Andersen Health Behaviour Model: predisposing, enabling, needs and personal health behaviour. The classifications from the MDC database were kept for most of the variables. The following variables were created from the existing ones or were reclassified.

**Table 1:** Characteristics of all women and women who entered ANC late

Variables	All women, N = 85,034		Women entering ANC after 12 weeks, n = 34,507	
	
	***n***	***% by column***	***n***	***% by row#***
**PREDISPOSING CHARACTERISTICS**				
Age*				
Teens	3,342	3.9	1,873	56
Twenties	35,359	42	15,954	45
Thirties	43,311	51	15,568	36
Forties and fifties	3,006	3.5	1,109	37
Missing	16	0.02	3	19
Race/Origin*				
Non Aboriginal Australian born	59,275	70	20,747	35
Aboriginal and Torres Strait Islanders	2,215	2.6	1,118	50
Migrants from developed countries	6,128	7.2	2,652	43
Migrants from developing countries	17,229	20	9,892	57
Missing	187	0.21	98	52

**ENABLING RESOURCE**				
Place of residence*				
Eastern Sydney	26,196	31	9,879	38
Western Sydney	29,382	35	15,929	54
Regional NSW	15,331	18	5,108	33
Rural/Remote NSW	12,086	14	2,858	24
Others states	2,039	2.4	733	36

**NEEDS**				
Number of previous pregnancies*				
Zero	36,270	43	14,325	40
One	28,605	33	10,901	38
Two	12,705	15	5,325	42
Three or more	7,415	8.7	3,941	53
Missing	39	0.05	15	38
Last delivery by caesarean*				
No, or no previous birth	74,841	88	31,052	41
Yes	10,105	12	3,423	34
Missing	88	0.15	32	36
***Baby sex@***				
Male	43,881	52	17,728	40
Female	41,121	48	16,766	41
Missing	32	0.04	13	41
***Complication during current pregnancy$***				
No	76,961	91	31,061	40
Yes	8,073	9.5	3,446	43
Number of foetus/es*				
Singleton	82,417	97	33,602	41
Twins, triplets or quadruplets	2,617	3.1	905	35

**HEALTH BEHAVIOUR**				
Number of cigarettes smoked per day*				
Non	73,491	86	28,690	39
Ten or less	6,375	7.5	2,902	46
More than ten	5,439	6.4	2,780	53
Missing	321	0.43	135	47

• # Proportion with this characteristic entering ANC after 12 weeks

• * indicates p values from bi-variate regressions <= 0.05

• @ indicates p values from bi-variate regressions > 0.05

• $ Pregnancy complication was significant in logistic regression (p < 0.05) but not in linear regression (p > 0.05).

#### Predisposing characteristics

Four categories of Race/origin (non Aboriginal Australian born, Aboriginal and Torres Strait Islander, migrant from developing countries and migrant from developed countries) were created from two existing variables in the MDC: Aboriginal/Torres Strait Islander status and country of birth.

#### Enabling resource

Area of residence was classified into five categories: East, North East and South East areas of Sydney are more affluent and thus were grouped into one area of Eastern Sydney. The West, South West and the far West areas of Sydney are less affluent and thus were grouped into one area called Western Sydney. Regional NSW includes the cities Newcastle, Wollongong and Gosford and the remaining towns are classified as rural and remote. Women whose place of residence was outside NSW were grouped into "Other states".

#### Needs

Women who had three or more previous pregnancies were grouped into one category because they were only a small number.

The number of previous caesarean deliveries was classified as yes or no because few women had more than one previous caesarean delivery.

The number of foetuses during previous pregnancies (not including this pregnancy) was classified into singleton or twins/triplets/quadruplets.

### Missing values

Records for women with missing pregnancy duration at entry to ANC were excluded from the analysis. For explanatory variables, missing categories were created so that all analyses have the same sample size.

### Regressions and model building

The previous studies have used linear regression [14,16] or logistic regression methods [11] or both [22] to identify factors related to pregnancy duration at first ANC visit (in month or week) and/or late entry to ANC.

We used both linear and logistic regression methods in this study, because the linear regression provides information on the proportion of variation in the pregnancy duration at entry to ANC explained by the contributing factors and the logistic regression provides information on the characteristics of women who entered ANC late. In addition, some factors may be significant in one model but not in the other. For example, if all women are aware that they should enter ANC within 12 weeks, there may be fewer factors that distinguish between entering ANC within 12 weeks or later, than factors that distinguish between entering ANC at 4 weeks, 8 weeks, 12 weeks, and later. Using both regression techniques increases the chance of a potential associated factor being identified. Furthermore; the guidelines may change in the future. For example, the US Public Health Service Expert Panel on Prenatal Care has recently recommended 8 weeks instead of 12 weeks [6]. Whenever the current standard guideline changes, the results from the linear regression will still be useful.

The outcome variable used in the logistic regression was classified into late entry to ANC (after 12 weeks, coded one) and early entry (within 12 weeks, coded zero). The outcome variable used in the linear regression was week of pregnancy.

For explanatory variables, number of women belonging to each category of variables was examined. Groups were chosen as reference groups (coded as 1 – lowest) if they had the largest number of women.

Bi-variate regression analyses were conducted to investigate the relationships between each of the individual factors and the outcomes. Variables with p value from bi-variate regressions equal to or less than 0.25 were considered in the next stage of the model building process.

Correlations between these variables were then examined. If there were high correlations between some variables, one of the variables in each pair would be considered to be omitted. The more relevant variable (found in the literature or viewed by researcher as to be more important) would be chosen to represent the other.

Stepwise selection technique was used in the model building process. The exclusion value for p was ≥ 0.10. Interactions between variables were examined. Significant variables in the logistic regression model were included in the linear regression model and vice versa, even when they were significant in only one model.

Blocks of variables were entered one after another into the models to determine the relative contribution of each block to the models.

### Statistical software

The MDC 2004 data was downloaded using SAS and transferred to STATA 8.0 [24].

## Results

A total of 85,626 women gave birth in NSW in 2004. Of these there were 85,034 women or 99% with data on pregnancy duration at entry to ANC.

### Characteristics of women

The second and third columns of Table 1 present characteristics of women who gave birth in NSW in 2004. The majority of women were in their twenties and thirties (42% and 51% respectively, mean age was 30 years), non Aboriginal Australian born (70%), living in Sydney (31% in Eastern and 35% in Western Sydney), having no or one previous pregnancy (44% and 34% respectively), having no previous caesarean section (88%), having no pregnancy complication (91%), having singleton current pregnancy (97%) and being non smokers (86%). The numbers of male and female babies born to these women were almost equal (52% for male and 48% for female).

### Pregnancy duration at entry to ANC

Table 2 shows pregnancy duration at entry to ANC. There was a significant number of women who entered ANC late by any definition: 41% entered ANC after 12 weeks, 16% after 17 weeks and 10% after 20 weeks. The mean pregnancy duration at entry to ANC was 12.8 weeks and the median duration was 12 weeks.

**Table 2:** Classification of pregnancy duration at entry to ANC, n = 85,034

	Frequency	Percentage (95%CI)
***Developed country guideline***		
Within 12 weeks	50,527	59.4% (59.1–59.8%)
After 12 weeks	34,507	40.6% (40.2–40.9%)

***WHO guideline***		
Within 17 weeks	71,108	83.6% (83.4%–83.8%)
After 17 weeks	13,926	16.4% (16.1–16.6%)

***NSW classification***		
Within 20 weeks	76,678	90.2% (90–90.4%)
After 20 weeks	8,356	9.8% (9.6–10%)

***Mean (weeks)***	12.8	12.7–12.8
***Median (weeks)***	12	

### Bi-variate analyses

The last two columns of Table 1 present the number and the proportions of women within each category entering ANC after 12 weeks. Younger women tended to enter ANC later than older women. For example, 56% of women in their teens entered ANC late while only 36% of women in their 30's entered ANC late. Other high risk groups were migrants from developing countries (57%), Aboriginal and Torres Strait Islanders (50%), women living in Western Sydney (54%), women having three or more previous pregnancies (53%) and heavy smokers (53%).

The bi-variate logistic and linear regression analyses showed that most of the variables were statistically significant (p < 0.05), except the sex of the baby (p > 0.05 in both logistic and linear regressions) and pregnancy complication (significant in logistic regression but not in linear regression).

### Correlation

Most of the variables were weakly correlated to each other, thus there was no strong evidence to exclude any variable. For example, migrants from developed countries were more likely to live in Eastern Sydney and migrants from developing countries were more likely to live in Western Sydney, however the correlations were all less than 0.25.

### Multivariate regression models

All variables except the sex of the baby were included in the initial full models. Obstetric complication was later excluded during the model fitting processes for both logistic and linear regressions. Interactions between number of previous pregnancies and last caesarean delivery, between residence and race/origin were added to the models but were not found to be significant.

Table 3 presents the reduced multivariate logistic regression model (the linear regression model is not presented). In the linear regression model, 12.5% of the variation in pregnancy duration at entry to ANC was explained by contributing factors. Predisposing variables and enabling resource contributed more to the model (r^2 ^= 5.6% and r^2 ^= 3.8% respectively) than needs (r^2 ^= 2.5%). Health behaviour contributed least (r^2 ^= 0.6%). Similar to the results from the linear regression, predisposing characteristics and enabling resource were also more significant (χ^2^_(df = 8) _= 2928, p < 0.001 and χ^2^_(df = 3) _= 2974, p < 0.001 respectively) than needs (χ^2^_(df = 7) _= 949, p < 0.001) and health behaviour was also least significant (χ^2^_(df = 3) _= 689, p < 0.001).

**Table 3:** Reduced regression models of pregnancy durationat entry to ANC, N = 83,034

Variables	Logistic regression#			
	
	***OR***	***95% CI***		***P>z***
**PREDISPOSING CHARACTERISTICS**				
***Age***				
Teens	2.99	2.76	3.23	<0.001
Twenties	1.59	1.54	1.64	<0.001
Thirties (reference)				
Forties and fifties	0.91	0.84	0.99	0.022
Missing	0.66	0.18	2.44	0.536
***Race/Origin***				
Non Aboriginal Australian born (reference)				
Aboriginal and Torres Strait Islanders	1.55	1.42	1.71	<0.001
Migrants from developed countries	1.47	1.39	1.56	<0.001
Migrants from developing countries	2.18	2.10	2.26	<0.001
Missing	2.12	1.57	2.86	<0.001

**ENABLING**				
***Residence***				
Eastern Sydney (reference)				
Western Sydney	1.57	1.51	1.63	<0.001
Regional NSW	0.76	0.73	0.80	<0.001
Rural/Remote NSW	0.41	0.39	0.43	<0.001
Others states	0.84	0.76	0.92	<0.001

**NEEDS**				
***Number of previous pregnancies***				
Zero (reference)				
One	1.12	1.08	1.16	<0.001
Two	1.38	1.32	1.44	<0.001
Three or more	2.17	2.05	2.30	<0.001
Missing	1.90	0.93	3.92	0.080
***Last caesarean delivery***				
No (reference)				
Yes	0.76	0.73	0.80	<0.001
Missing	0.83	0.51	1.35	0.458
***Number of foetuses***				
Singleton (reference)				
Twins Triplets or quadruplets	0.86	0.79	0.94	0.001

**HEALTH BEHAVIOUR**				
***Number of cigarettes per day***				
Non (reference)				
Ten or less	1.39	1.32	1.47	<0.001
More than ten	1.85	1.74	1.96	<0.001
Missing	1.51	1.18	1.92	0.001

**Constat**	-	-	-	-

**Model Chi square (df)(p)**	8729 (22) (<0.001)			

**Model F test (df) (p)**				

# Outcome was either entered ANC early (within 12 weeks) or late (after 12 weeks)

There were also agreements between the two models on the significances of factors investigated. All of the significant factors in the logistic regression model were significant in the linear regression and vice versa. The factors related to late entry to ANC were younger age, being a migrant, being Aboriginal or Torres Strait Islander, living in Western Sydney, having more previous pregnancies and a history of smoking. The groups of women at highest risk were teenagers (OR = 2.99, 95%CI = 1.76–3.23), migrants from developing countries (OR = 2.18, 95%CI = 2.1–2.26), women with three or more previous pregnancies (OR = 2.17, 95%CI = 2.05–2.30), heavy smokers (OR = 1.85, 95%CI = 1.74–1.96) and women living in Western Sydney (OR = 1.57, 95%CI = 1.51–1.63). The largest groups of women at high risk were migrants from developing countries (n = 9,892) and women living in Western Sydney (n = 29,382). The results from the two models were similar in terms of the level of risk. For example, the highest risk group (teenagers) had the highest OR (2.99) and the highest coefficient (3.6).

The factors associated with a reduced risk of late entry to ANC were age 40 and older, living outside of Sydney, having previous caesarean delivery and having a multiple pregnancy.

## Discussion

The study attempted to assess the prevalence of women who entered ANC late in NSW in 2004 and identify related factors. Forty one percent of women entered ANC after 12 weeks of gestation. This was double the 1997–1998 Victorian figure of 20% [8]. Over the last six years in NSW there has been a small reduction in the proportion of women entering ANC after 20 weeks of gestation in NSW (10% in this study compared to 13% in 1998 [9]). A significant proportion of women (16%) failed to meet the developing countries' recommendation of entering ANC in the first four months of gestation [1].

The fact that predisposing characteristics and enabling resources accounted more for the variation in pregnancy duration at entry to ANC than needs, implied that inequality existed between women. Inequalities between women were also found in other studies that utilised the Andersen Health Behaviour Model [11,12,14,25]. Our results are also similar to the findings from other studies on the significance of age [11], race [13,18] and parity [11-13,15]. Younger women, especially teenagers, are more likely to have unplanned pregnancies and lack information and the resources to access ANC services. Aboriginal and Torres Strait Islander women may have less knowledge about ANC and may have traditional beliefs that delay entering ANC. Women from developing countries bring with them the knowledge and practice from their first home countries and these are often lower in standards than more developed countries. Women with more previous pregnancies may be more confident because of their experience. They may also find it harder to attend ANC because of difficulties with child care.

Some other significant factors in this study have not been found to be related to late entry to ANC but have been found to be related to overall ANC utilisation (which included pregnancy duration at entry to ANC and number of ANC visits). These factors were previous caesarean delivery [22] and smoking [26]. Women who have had a previous caesarean delivery are aware of the risk of pregnancy complications and therefore more likely to seek ANC early. Smokers practice less healthy care behaviours in general and in ANC in particular.

Although similar to findings from other studies in the world, some results were unique for Australia. Migrants from developing and from developed countries entered ANC later than women who were born in Australia. Migrants may have to focus on other more personal or family priorities such as stable accommodation and employment. Being new to the country means lack of information and support. In addition, these women may not have health insurance and are not entitled to social welfare benefits if they do not meet certain residency requirements.

In contrast to other studies that found living in more developed areas, mostly metropolitan areas, increases early ANC attendance [10-12], this study found that living in Sydney, especially in Western Sydney is related to late entry to ANC, despite the fact that Sydney is the capital of the state. The explanation could be that most of the migrants live in Sydney and very few migrants live outside of Sydney. The other reason could be due to the effect of the Family First Program, introduced by the NSW Health Department to improve women's and children's health. ANC is one of the components of the Program in the rural and remote areas, and in Aboriginal communities [27].

The strength of the study was that most of the women in NSW who gave birth in 2004 were included – reflecting the real situation in NSW. However, due to the large number of women, small differences were likely to be detected as statistically significant. Information was recorded by health staff throughout women's pregnancy, therefore data accuracy was at a high level.

This is the first study in Australia that utilised the widely used Health Seeking Behaviour Model in the selection and analysis of factors related to late entry to ANC.

The main limitation of the study was that several potential important factors such as health insurance, income and difficulty in transportation were not available for analyses. This could be one of the reasons for the linear regression model to explain only 12.5% of the variation.

## Conclusion and recommendations

In 2004, a large number of NSW women entered ANC later than international and national recommendations and inequality existed between subgroups of women. For effective use of resources, efforts to increase early entry to ANC should focus on the identified high risk groups of women, especially the groups with large number of women such as migrants from developing countries and women living in Western Sydney. Interventions should be tested before large scale application.

Future research in Australia should include other potential explanatory factors and expand to other aspects of ANC such as number of ANC visits, services provided during ANC visits and satisfaction of women and providers.

## Competing interests

The author(s) declare that they have no competing interests.

## Authors' contributions

LTTT designed the study, downloaded and analysed the data, drafted the manuscript. GR participated in the designing of the study and drafting of the manuscripts. All authors read and approved the final manuscript.

## References

[B1] WHO (1994). Antenatal Care. Report of a Technical Working Group, 1994 - WHO/FRH/MSM/968.

[B2] http://www.nice.org.uk/page.aspx?o=CG006 , National Institute for Health and Clinical Excellence (2003). Antenatal care: Routine care for healthy pregnant women.

[B3] ed , American Academy of Pediatrics (2002). Guidelines for perinatal care.

[B4] Carroli G, Villar J, Piaggio G, Khan-Neelofur D, Gulmezoglu M, Mugford M, Lumbiganon P, Farnot U, Bersgjo P, W. H. O. Antenatal Care Trial Research Group (2001). WHO systematic review of randomised controlled trials of routine antenatal care.[comment]. Lancet.

[B5] Villar J, Ba'aqeel H, Piaggio G, Lumbiganon P, Miguel Belizan J, Farnot U, Al-Mazrou Y, Carroli G, Pinol A, Donner A, Langer A, Nigenda G, Mugford M, Fox-Rushby J, Hutton G, Bergsjo P, Bakketeig L, Berendes H, Garcia J, W. H. O. Antenatal Care Trial Research Group (2001). WHO antenatal care randomised trial for the evaluation of a new model of routine antenatal care.[comment][erratum appears in Lancet 2001 Nov 3;358(9292):1556]. Lancet.

[B6] Mortimer GRMDIRMMDJGHBA (1991). Caring for our future: a report by the expert panel on the content of prenatal care. Obstet Gynecol.

[B7] Hunt JM, Lumley J (2002). Are recommendations about routine antenatal care in Australia consistent and evidence-based?. The Medical Journal of Australia 18 March 2002 176 6: 255-259.

[B8] Halliday J, Ellis I, Stone C (1999). WUDWAW: Who Usually Delivers Whom and Where: report on models of antenatal care. Victorian Perinatal Data Collection Unit, Department of Human Services.

[B9] Taylor L, Pym M, Bajuk B, Sutton L, Trvis S, Banks C (2000). New South Wales Mothers and Babies 1998. Public Health Division, NSW Health Department.

[B10] McDonald TP, Coburn AF (1988). Predictors of prenatal care utilization. Social Sciences & Medicine.

[B11] Perloff JD, Jaffee KD (1999). Late entry into prenatal care: The neighborhood context. Social work.

[B12] Trinh LTT (2005). Antenatal care in three provinces in Vietnam: Long an, Ben tre and Quang ngai. Ph.D thesis. Centre for Clinical Epidemiology and Biostatistic.

[B13] Magadi MA, Madise NJ, Rodrigues RN (2000). Frequency and timing of antenatal care in Kenya: explaining the variations between women of different communities. Social Science & Medicine.

[B14] LaVeist TA, Keith VM, Gutierrez ML (1995). Black/white differences in prenatal care utilization: an assessment of predisposing and enabling factors. Health Services Research.

[B15] Navaneetham K, Dharmalingam A (2002). Utilization of maternal health care services in Southern India. Social Science and Medicine.

[B16] Ivanov LL (2000). Use of a Western theoretical model to investigate the relationships among characteristics of pregnant women, utilization, and satisfaction with prenatal care services in St. Petersburg, Russia. Public Health Nursing.

[B17] Gazmararian JA, Arrington TL, Bailey CM, Schwarz KS, Koplan JP (1999). Prenatal care for low-income women enrolled in a managed-care organization. Obstetrics & Gynecology.

[B18] NSW Health Department (1997). NSW Mothers and Babies Report 1997. Part 4: Maternal country of birth. http://internalhealthnswgovau/public-health/mdc97/4_14htm.

[B19] Andersen RM (1968). Behavioral Model of Families' Use of health Services. Chicago, IL, University of Chicago, Center for Health Administration Studies Research Series.

[B20] Andersen RM (1995). Revisiting the Behavioral Model and Access to Medical Care: Does it Matter. Journal of Health and Social Behavior.

[B21] Phillips KA, Morrison KR, Andersen RM, Aday LA (1998). Understanding the context of health care utilization: assessing environmental and provider related variables in the behavioral model of utilization. Health Services Research.

[B22] Glei DA (2003). Utilization of Care During Pregnancy in Rural Guatemala: Does Obstetrical Need Matters?. Social Science & Medicine.

[B23] NSW Health Department (2005). NSW midwives collection. http://wwwhealthnswgovau/public-health/mdc/mdc95html.

[B24] StataCorp (2004). Stata Statistical Software: Release 8.2.

[B25] Ivanov LL, Flynn BC (1999). Utilization and satisfaction with prenatal care services. Western Journal of Nursing Research.

[B26] Petrou S, Kupek E, Vause S, Maresh M (2001). Clinical, provider and sociodemographic determinants of the number of antenatal visits in England and Wales. Social Science & Medicine.

[B27] Family First NSW (2005). http://wwwfamiliesfirstnswgovau.

